# Risk factors for neurocognitive impairment and the relation with structural brain abnormality in children and young adults with severe chronic kidney disease

**DOI:** 10.1007/s00467-022-05781-1

**Published:** 2022-11-02

**Authors:** Sophie Lijdsman, Kim J. Oostrom, Marit S. van Sandwijk, Antonia H. Bouts, Koen van Hoeck, Huib de Jong, Jaap Oosterlaan, Frederike J. Bemelman, Marsh Königs, Jaap W. Groothoff

**Affiliations:** 1 Department of Child and Adolescent Psychiatry & Psychosocial Care, Amsterdam Reproduction & Development, Emma Children’s Hospital, Amsterdam University Medical Centers (Amsterdam UMC), University of Amsterdam, G8-136, PO Box 22660, 1100 DD Amsterdam, The Netherlands; 2 Department of Nephrology, Amsterdam Infection & Immunity, Amsterdam UMC, University of Amsterdam, Amsterdam, The Netherlands; 3 Dianet Dialysis Centre, Amsterdam, The Netherlands; 4 Department of Pediatric Nephrology, Amsterdam Reproduction & Development, Emma Children’s Hospital, Amsterdam UMC, University of Amsterdam, Amsterdam, The Netherlands; 5 Department of Pediatrics, University of Antwerp, Antwerp, Belgium; 6 Department of Pediatrics, Sophia Children’s Hospital, Erasmus MC, Rotterdam, The Netherlands; 7 Emma Neuroscience Group, Department of Pediatrics, Amsterdam Reproduction & Development, Emma Children’s Hospital, Amsterdam UMC, University of Amsterdam, Amsterdam, The Netherlands

**Keywords:** Cognition, Executive functioning, Diffusion tensor imaging, Kidney replacement therapy, Brain structure

## Abstract

**Background:**

Severe chronic kidney disease (CKD) in children and young adults has shown to be associated with abnormal brain development, which may contribute to neurocognitive impairments. We aimed to investigate risk factors for neurocognitive impairment and investigate the relation with structural brain abnormalities in young severe CKD patients.

**Methods:**

This cross-sectional study includes 28 patients with severe CKD (eGFR < 30), aged 8–30 years (median 18.5 years), on different treatment modalities (pre-dialysis [*n* = 8], dialysis [*n* = 8], transplanted [*n* = 12]). We assessed neurocognitive functioning using a comprehensive test battery and brain structure by magnetic resonance imaging metrics of brain volume and white matter integrity (fractional anisotropy [FA] and mean diffusivity [MD] measured with diffusion tensor imaging). Multivariate regression and mediation analyses were performed between clinical CKD parameters, brain structure, and neurocognitive outcome.

**Results:**

A combination of risk factors (e.g., longer time since kidney transplantation, longer dialysis duration and late CKD onset) was significantly associated with lower intelligence and/or worse processing speed and working memory. Lower FA in a cluster of white matter tracts was associated with lower intelligence and mediated the relation between clinical risk factors and lower intelligence.

**Conclusions:**

Young severe CKD patients with a prolonged duration of kidney replacement therapy, either dialysis or transplantation are at particular risk for impairments in intelligence, processing speed, and working memory. Disrupted white matter integrity may importantly contribute to these neurocognitive impairments. Prospective, longitudinal studies are needed to elucidate the mechanisms involved in CKD and treatment that affect white matter integrity and neurocognitive outcome in young patients.

**Graphical abstract:**

**Figure:**
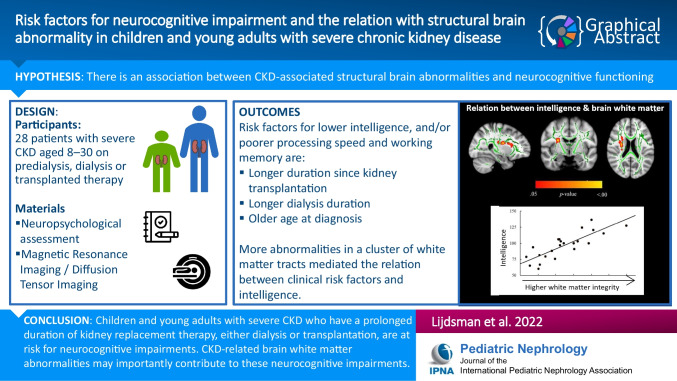
A higher resolution version of the Graphical abstract is available as Supplementary information

**Supplementary information:**

The online version contains supplementary material available at 10.1007/s00467-022-05781-1.

## Introduction

Young patients with advanced stages of chronic kidney disease (CKD) are at particular risk for school and vocational problems, which have partly been attributed to impaired neurocognitive functioning associated with CKD [1–3]. CKD-associated neurocognitive dysfunction may be expressed in impaired processing speed, attention, memory and executive functioning [2, 4]. Likewise, severe CKD is suggested to be associated with abnormal brain development (e.g., due to exposure to dialysis treatment, hypertension, and neurotoxic and vasoactive immunosuppressive therapy after kidney replacement therapy (KRT) [5–9]), which may play an important role in the development of these neurocognitive impairments. Recently, we showed that children and young adults with severe CKD are at risk for widespread disruption of white matter integrity and regional loss of subcortical volume (i.e., in nucleus accumbens) [10], but the association between such abnormalities and neurocognitive functioning in young patients with severe CKD has remained largely unexplored.

In adults and elderly with advanced stages of CKD, both lower white matter integrity and smaller brain volumes have been related to impairments in global neurocognitive functioning, attention, and executive functioning [11–16]. In children and young adults with CKD, studies have reported on the association between structural brain abnormalities and neurocognitive functioning in children with mild to moderate stages of CKD [17, 18] or patients up to 25 years old with mild to severe stages of CKD [19]. These studies yielded inconsistent findings and did not find clear evidence for an association between CKD-associated brain abnormalities and neurocognitive functioning. To date, the potential relevance of brain volume and white matter integrity for neurocognitive functioning has not been investigated in children and young adults with advanced CKD stages receiving KRT. Given the vulnerability of the developing brain for diffuse impact [20, 21] and the potential impact on daily life functioning, better understanding of the relation between severe CKD-associated brain abnormalities and neurocognitive functioning in young patients is of great importance.

The current study aims to explore the potential role of structural brain abnormalities in the relationship between advanced CKD, different treatment modalities, and neurocognitive functioning (i.e., intelligence and specific neurocognitive functions).

## Methods

### Participants

Twenty-eight CKD patients with severe CKD were recruited from the Amsterdam University Medical Center (Amsterdam UMC, *n* = 25); Erasmus Medical Center, the Netherlands (*n* = 1); and the University Hospital Antwerp, Belgium (*n* = 2). Inclusion criteria were as follows: (1) CKD stage 4–5 (estimated glomerular filtration rate [eGFR] < 30 ml/min/1.73 m^2^) on conservative therapy, on peritoneal or hemodialysis or patients having received a kidney transplant at least 2 years prior to enrollment (to ensure stable kidney function), and (2) aged between 8 and 30 years. Exclusion criteria were as follows: (1) previously established severe intellectual impairment; (2) insufficient mastery of the Dutch language; (3) primary sensory disorder (hearing or vision impairments); (4) established skull or brain abnormalities not related to CKD; or (5) co-existing disease with primary or secondary central nervous system involvement interfering with the impact of CKD.

#### Treatment subgroups

The following treatment subgroups were distinguished: (1) a pre-dialysis group (*n* = 8) with eGFR < 30 ml/min/1.73 m^2^ on conservative treatment at time of assessment; (2) a dialysis group (*n* = 8); and (3) a transplanted group (*n* = 12) of patients with a stable, functioning kidney graft for at least 2 years and eGFR > 30 ml/min/1.73 m^2^.

### Measurements

#### Socio-demographic

Socio-demographic characteristics (i.e., age, sex, parental educational level) were collected using a custom-made questionnaire assessed through the online KLIK portal [22]. Parental educational level was divided into three categories: (1) low education (primary education, lower vocational education, lower and middle general secondary education); (2) middle education (middle vocational education, higher secondary education, pre-university education); and (3) high education (higher vocational education, university) [23].

#### Clinical CKD parameters and previous MRI outcomes

Clinical CKD parameters were extracted from the patient’s medical file and included: age at diagnosis of severe CKD, current eGFR, duration of severe CKD (ratio of time interval between date at which eGFR < 30 ml/min/1.73 m^2^ and assessment date to calendar age, expressed as % of life), dialysis duration (ratio of dialysis duration to calendar age, i.e., % of life), and time since successful transplantation (ratio of time interval between date of transplantation and eGFR > 30 ml/min/1.73 m^2^ and assessment date to calendar age, i.e., % of life).

Brain structure was assessed by MRI, using diffusion tensor imaging with tract-based spatial statistics to determine white matter integrity and using segmentation-based volumetric analysis on T1 scans to determine brain volumes. Details on the MRI acquisition and preprocessing are described elsewhere [10]. For this study, only brain measures with observed differences between our CKD patients (*n* of MRI patient sample = 23) and a control group were used. This involved white matter integrity in a large cluster of white matter tracts (*i.e.*, lower fractional anisotropy [FA; value ranging from 0.0 to 1.0] and higher mean diffusivity [MD], mainly in the inferior frontal occipital fasciculus [IFOF], anterior thalamic radiation [ATR], superior longitudinal fasciculus [SLF], cortical spinal tract [CST], and uncinate fasciculus) and smaller volume in a subcortical gray matter structure (i.e., nucleus accumbens).

#### Neurocognitive functioning

A short form of the Wechsler (Adult) Intelligence Scale (WISC/WAIS, third edition) was used to estimate age-standardized full-scale intelligence quotient (eFSIQ; *M* = 100, *SD* = 15) and interpreted based on clinical interpretation guidelines [24]. A comprehensive neurocognitive test battery was administered to assess a wide range of specific neurocognitive functions (see Table 1). For these neurocognitive tests, age-standardized scaled scores were calculated (e.g., by comparing the patients’ individual score to normative data) for each participant and subsequently transformed to z-scores where lower scores correspond to worse performance.

**Table 1:** Comprehensive neuropsychological test battery: neurocognitive measures and neurocognitive tasks used

Neurocognitive measures	Description of neurocognitive measures	Description of neurocognitive task and condition
Estimation of age-standardized full-scale intelligence quotient (eFSIQ)	Estimation of intellectual capacities	eFSIQ (*M* = 100, *SD* = 15) was assessed using Wechsler Intelligence Scale short forms (WISC-III-NL [42] for patients < 17 y, WAIS-III-NL [43] for patients > 17 y) consisting of combined age-scaled scores of Vocabulary and Block Design subtests, which has shown high validity and reliability in estimating IQ [44]. In the Vocabulary subtest, patients had to explain the meaning of words and in the Block Design subtest, patients had to construct a visuo-spatial figure with blocks
Working memory	The ability to memorize verbal information for a short time and manipulate it	Age-scaled score of Digit-Span subtest (WISC-III-NL/WAIS-III-NL), where patients had to repeat a sequence of verbally presented numbers (increasing in length) in either forward, backward, or ascending order (WAIS-III-NL only)
Memory encoding	The ability to learn new information	Total age-scaled score of the Dutch version of Rey’s Auditory Verbal Learning Test (RAVLT) [45, 46], where fifteen words were verbally presented for 5 times. Each time patients had to remember as many words as possible. The total score consists of the total amount of words patients remembered during this repeated presentation
Memory consolidation–retrieval	The ability to store information in long-term memory and recall information from long-term memory	Delayed recall age-scaled score of RAVLT (corrected for total score), which is the amount of words patients remember after 25 min
Visual scanning	The speed to locate visual information	Age-scaled score on visual scanning condition of Trail Making Test, of Delis-Kaplan Executive Function System (D-KEFS) [47]. In this condition, a large amount of varying numbers and letters is presented visually on a large paper sheet and patients need to indicate one single number (i.e., number 3) as quickly as possible
Motor speed	The speed of basic visuo-motor functioning	Age-scaled score on motor speed condition of Trail Making Test (D-KEFS) [47], where patients had to connect small circles in a pre-assigned way on a large paper sheet as quickly as possible
Sequencing	The speed of basic mental sequencing	Combined age-scaled scores of the digit sequencing and letter sequencing conditions of Trail Making Test (D-KEFS) [47], where patients had to respectively sequence visually presented numbers in ascending order and sequence letters in alphabetical order as quickly and accurate as possible
Visuo-motor switching	The ability to mentally switch between different mental concepts by using visuo-motor functions	Age-scaled score of the switching condition controlled for the combined sequencing score of Trail Making Test (D-KEFS) [47]. In the Trail Making Test switching condition, patients had to alternate between numbers and letters while connecting them in ascending and alphabetical order as quickly and accurate as possible
Speed	The speed of basic mental processing and verbal responses	Combined age-scaled scores of the naming and reading condition of Color Word Interference (D-KEFS) [47], which is a more verbal task where patients have to respond verbally to visually presented information. The speed measure consists of the naming condition (i.e., patients had to name the color of colored squares as quickly as possible) and the reading condition (i.e., patients had to read spelled out colors presented in black ink as quickly as possible)
Interference control	The ability to inhibit an automatic response	Age-scaled score of the interference control condition controlled for naming condition of Color Word Interference (D-KEFS) [47], where colors are written down printed in incongruent ink color. Patients have to name the name the color of the ink as quickly and accurately as possible
Verbal switching	The ability to mentally switch between different mental concepts by verbally responding to visual information	Age-scaled score of the switching condition controlled for interference control condition of Color Word Interference (D-KEFS) [47], where colors are again written down in incongruent ink color. In the switching condition, some words are presented with a small rectangle around it and patients are asked to read aloud the words surrounded with a rectangle, but to name the ink color for words without a rectangle
Category fluency	The ability and speed to access verbal information based on semantics	Age-scaled score of the Word Production subtest (NEPSY-II-NL) for patients < 12 y [48] and Category fluency task for patients > 12 y [49], where patients had to name as many words as possible during 1 min within a specific category (i.e., animals and food/drinks if < 12 y, and supermarket articles and professions if > 12 y)
Letter fluency	The ability and speed to access verbal information based on phonemes	Age-scaled score of the Word Production subtest (NEPSY-II-NL) for patients < 12y [48] and Letter word fluency task for patients > 12 y [50], where patients had to name as many words as possible during 1 min beginning with a specific letter (i.e., S/M if < 12 y, or D/A/T if > 12 y)

D-KEFS = Delis-Kaplan Executive Function System; eFSIQ: Estimation of age-standardized full-scale intelligence quotient; NEPSY-II-NL = A Developmental NEuroPSYchological Assessment; RAVLT = Rey’s Auditory Verbal Learning Test; WISC-III = Wechsler Intelligence Scale for Children – Third Edition; WAIS-III = Wechsler Adult of Intelligence Scale – Third Edition

To reduce the number of neurocognitive outcome measures, data reduction was performed using principal component analyses with Varimax rotation on the age-standardized z-scores derived from the neurocognitive tasks (see Supplement 1 for specifications). The principal component analyses constructed five domains, accounting for 76% of explained variance. Based on factor loadings the neurocognitive domains were labeled as follows: (1) processing speed and working memory; (2) fluency; (3) verbal memory; (4) processing speed, switch, and control; (5) switching.

### Procedure

The study protocol was approved by the Medical Ethics Committee of the Amsterdam UMC (#NL61708.018.17), and all procedures were performed according to the Declaration of Helsinki. Eligible patients were first approached by their treating physician. Interested participants were contacted by a member of the research team and received a comprehensive information letter. After 2 weeks, potential candidates were re-contacted by telephone to answer any possible remaining questions. Written informed consent was obtained from legal guardians (for children aged < 16 years old) and/or children or young adults aged ≥ 12 years.

The 30-min MRI scanning and 90-min neurocognitive assessments (administered in a dedicated assessment room by a trained neuropsychologist using standardized instructions) were performed on the same day and took place at the Amsterdam UMC. One week earlier, participants and/or parents of participants < 18 years old completed the online questionnaires.

### Statistical analyses

#### Socio-demographic and clinical characteristics

Statistical analyses were performed using SPSS 26.0 (IBM Corp., 2019). Independent and dependent variables were tested for normality and screened for outliers (± 3 interquartile ranges below/above the lower or upper quartile), which were rescaled using Winsorizing [25]. Missing data in the neurocognitive outcomes (*M* = 2%, range: 0–7%) were replaced by multiple imputation [26]. All treatment subgroups were compared with each other on demographic characteristics (age, sex, and parental educational level) and clinical CKD parameters using analysis of variance (ANOVA), to enable subgroup comparisons on outcome variable. Multivariate regression models were checked for multicollinearity. Socio-demographic characteristics (i.e., age, sex and parental educational level) were added as covariates to all analyses.

#### Neurocognitive functioning

First, univariate linear regression analyses were performed for each clinical CKD parameter (age at diagnosis of severe CKD, current eGFR, severe CKD duration, dialysis duration, and time since successful transplantation). Then, the relationships between pre-selected clinical CKD parameters (with *p* < 0.20 in univariate analyses) and neurocognition were investigated using multivariate linear regressions with backward elimination (in order to account for suppressor effects [25], criterion for removal: *p* > 0.10). The neurocognitive outcome measurements were used as dependent variables (eFSIQ and neurocognitive domains) in multivariate linear regressions. Additionally, explorative analyses using ANOVA tested possible differences in neurocognitive functioning between treatment subgroups (pre-dialysis group, dialysis group, transplanted group).

#### Brain structure and neurocognitive functioning

We investigated the relation between brain structure and neurocognitive functioning using several steps. First, we aimed to determine the clusters of white matter tracts that are related to neurocognitive outcomes with observed sensitivity to CKD (i.e., neurocognitive outcomes that were significantly related to clinical CKD parameters in the analyses described above). This was performed using voxel-wise regression between these neurocognitive outcomes and FA within a cluster of white matter tracts that has previously been shown to be affected by CKD in this sample [10]. A voxel-wise approach was chosen to analyze white matter integrity, as it was expected that the impact of CKD on white matter integrity is diffuse and not limited to pre-specified regions of interest. FSL 5.0.11 *randomize* was used, where threshold-free cluster enhancement and family-wise error corrected *p*-values accounted for multiple testing. Any resulting clusters of white matter tracts with a significant relationship between FA and neurocognitive outcome were selected as regions of interest (ROI) for subsequent multivariate analysis.

Next, multivariate analyses on the relation between brain structure and neurocognitive functioning were performed in SPSS. Univariate linear regression analyses were performed for each aspect of brain structure with previously determined sensitivity for CKD in this sample (i.e., FA and MD in ROIs as determined in the previous step, and subcortical volume of the nucleus accumbens as determined in previous work on this sample [10]) on the neurocognitive outcomes with observed sensitivity to CKD using the multivariate regressions with backward elimination. From these univariate analyses, pre-selected aspects of brain structure (*p* < 0.20) were entered in multivariable linear regression models with backward elimination on neurocognitive outcomes with observed sensitivity to CKD. Finally, the potentially mediating role of brain structure in the relation between CKD and neurocognitive functioning was explored using mediation models with PROCESS version 4.0 using 5000 bootstrap samples [27].

All stat﻿istical testing was two-sided and alpha was set at 0.05. Cohen’s *d* effect sizes are reported where appropriate and were interpreted as small (*d* < 0.5), medium (0.5 ≥ *d* < 0.8), or large (*d* ≥ 0.8) [28].

## Results

### Socio-demographic and clinical characteristics

Age at diagnosis was significantly higher in the dialysis group than in the transplanted group (*p* = 0.010,* d* = 1.34). Treatment subgroups differed significantly in terms of eGFR, blood urea, and time since successful transplantation (Table 2).

**Table 2:** Demographic and clinical characteristics in the CKD and treatment subgroups

	CKD group	CKD treatment group^a^			Statistics	
		Pre-dialysis	Dialysis	Transplanted	*p*	contrasts
*n*	28	8	8	12		
Age (years)	18.5 (9.1–30.5)	15.7 (9.1–26.6)	23.0 (9.6–27.1)	17.8 (9.1–30.5)	0.192	
Male, *n* (%)	*n* = 18 (64%)	*n* = 6 (75%)	*n* = 4 (50%)	*n* = 8 (67%)	0.595	
Educational level of parents^b^	2.0 (1.0–3.0)	2.0 (2.0–3.0)	2.0 (1.0–3.0)	2.0 (1.0–3.0)	0.493	
Age at CKD diagnosis (years)	14.3 (0.0–24.7)	14.6 (1.9–24.7)	19.9 (9.3–24.0)	8.1 (0.0–22.8)	**0.030**	D > Tx
Primary disease^c^						
CAKUT^1^	8	3	0	5		
Renovascular^2^	3	0	0	3		
Cortical necrosis^3^	3	0	2	1		
Acquired glomerulopathy^4^	4	2	2	0		
Inherited nephropathy^5^	7	2	3	2		
Other and unknown cause^6^	3	1	1	1		
History of relevant comorbidities						
Extreme prematurity (< 32 weeks of GA)	1	0	0	1		
Malignant hypertension^d^	5	0	2	3		
Convulsions/history of epilepsy	2	0	1	1		
eGFR (ml/min/1.73 m^2^)^e^	26.6 (10.0–90.0)	23.7 (11.3–29.0)	10.0 (10.0–10.0)	51.6 (31.0–90.0)	**< 0.001**	Tx > PD and D
Urea (mmol/l)^f^	14.9 (5.1–28.8)	16.2 (13.7–20.7)	21.4 (16.8–28.8)	8.0 (5.1–14.5)	** < 0.001**	D > PD > Tx
Duration severe CKD (% of life)	12% (0–81%)	7% (0–81%)	11% (3–45%)	20% (4–78%)	0.585	
Ever treated by dialysis (*n*)	16	3	8	5		
Hemodialysis	7	3	3	1		
Peritoneal dialysis	8	–	5	3		
Both	1	–	–	1		
Duration dialysis (% of life)	1% (0–49%)	0% (0–6%)	4% (1–40%)	0% (0–49%)	0.391	
Ever treated by kidney transplantation (*n*)	16	2	2	12		
Pre-emptive	8	1	–	5		
Non-pre-emptive	8	1	2	7		
Time since kidney transplantation (% of life)	6% (0–75%)	0% (0–6%)	0 (0–10%)	23% (13–75%)	** < 0.001**	Tx > PD and D

Values are displayed as median (range), unless otherwise indicated. Abbreviations: CKD = chronic kidney disease; D = dialysis group; eGFR = estimated glomular filtration rate; GA = gestational age, PD = pre-dialysis group; Tx = transplanted group. Significant values (*p* < .05) are shown in bold.

^a^CKD patients who previously underwent a kidney transplantation, but had an eGFR<30 at time of assessment were allocated to either the pre-dialysis or dialysis group, according to their current treatment mode

^b^1.0 = low education, 2.0 = middle education, 3.0 = high education

Clinical CKD parameters were extracted from the patient’s medical file, specifications are as follows:

^c^Primary diseases: ^1^ urethral valves (*n *= 7), dysplasia (*n *= 1), ^2^ atypical hemolytic uremic syndrome (*n *= 1), malignant hypertension (*n *= 2),^3^ due to asphyxia (*n *= 1), due to septicemia (*n *= 2); ^4^ primary focal segmental glomerulosclerosis (*n *= 2), anti-neutrophilic cytoplasmic autoantibodies (ANCA) vasculitis (*n *= 1), LE-nephritis (*n *= 1); ^5^ branchiootorenal (BOR-) syndrome (*n *= 1), *NHPH1* mutation (*n *= 1), autosomal dominant polycystic kidney disease (ADPKD) (*n *= 1), Alport’s syndrome (*n *= 1), inherited FSGS due to *INF2* mutation (*n *= 2), *Pax-2* mutation (*n *= 1); ^6^ tubulointerstitial nephritis (*n *= 1), unknown cause (*n *= 2)

^d^malignant hypertension was defined as extremely high blood pressure resulting in organ damage

^e^ creatinine levels were obtained closest to the date of study participation (range: –51 days to +4 days relative to participation date), of which eGFR was calculated using Schwarz formula for patients aged <18 years and the abbreviated Modification of Diet in Renal Disease formula was used for patients aged >18. Due to large fluctuations in eGFR prior to and after dialysis, eGFR of patients receiving dialysis was conservatively set at 10

^d^urea blood levels were obtained closest to the date of study participation (range: –51 days to +4 days relative to participation date)

### Neurocognitive functioning in severe CKD patients

Univariate analyses in the total CKD group on eFSIQ showed *p*-values < 0.20 for dialysis duration and time since successful transplantation (Supplement 2). The final multivariate regression analysis revealed that longer time since successful kidney transplantation was significantly related to lower eFSIQ (Table 3), with parental educational level and dialysis duration included in the model as non-significant predictors.

**Table 3:** Multivariate regression models on associations between clinical CKD parameters and neurocognitive functioning

Outcome variable	Predictors retained in the model	Statistics			Model performance		
		*B (SE)*	*β*	*P*	*F*	*R*^*2*^	*p*
eFSIQ	Parental educational level	11.02 (6.02)	0.335	0.079	5.44	0.405	0.005
	Dialysis duration (% of life)	− 1.32 (0.74)	− 0.326	0.085			
	Time since kidney transplantation (% of life)	− 0.34 (0.15)	− 0.360	0.033			
Processing speed and working memory	Age at CKD diagnosis (years)	− 0.05 (0.02)	− 0.367	0.010	15.99	0.561	0.000
	Dialysis duration (% of life)	− 0.13 (0.03)	− 0.655	0.000			

Age, sex, and parental educational level were added as covariates to all analyses

Abbreviations: CKD = chronic kidney disease, eFSIQ = estimation of age-standardized full-scale intelligence quotient, SE = standard error

Univariate analyses on processing speed and working memory showed *p*-values < 0.20 for age at CKD diagnosis and dialysis duration. The final multivariate regression analysis revealed that higher age at CKD diagnosis and longer dialysis duration were significantly related to worse processing speed and working memory.

Univariate analyses on verbal memory showed *p*-values < 0.20 for duration of severe CKD and dialysis duration. The final multivariate regression analysis on verbal memory was not significant. Univariate analyses for other neurocognitive domains were not significant.

### Neurocognitive functioning in CKD treatment groups

Explorative analyses comparing treatment subgroups on neurocognitive functioning were not significant (Table 4). Based on clinical interpretation guidelines, exploratory visual inspection of eFSIQ and processing speed and working memory data shows that the pre-dialysis group scored in the high/above average range and both the dialysis and transplanted group scored in the low-average range. Therefore, we also explored differences in eFSIQ and processing speed and working memory domain between the group not receiving kidney replacement therapy (non-KRT: the pre-dialysis group) and the groups receiving kidney replacement therapy (KRT: dialysis and transplanted group combined) (see Supplement 3). These exploratory analyses showed that the non-KRT group had significant higher eFSIQ than the KRT group (*p* = 0.030, *d* = 0.99). The comparison on processing speed and working memory was not significant (*p* = 0.157, *d* =  − 0.94).

**Table 4:** Scores on neurocognitive domains in the pre-dialysis, dialysis, and transplanted group

	Treatment subgroup			Statistics^a^	
	Pre-dialysis	Dialysis	Transplanted	*p*	Covariates
*Neurocognitive domains*					
* n*	8	8	12		
eFSIQ	112 (12.5)	95 (21.8)	92 (21.4)	0.098	Parental educational level: *p* = 0.021
Processing speed and working memory domain	0.63 (0.69)	− 0.53 (1.26)	− 0.06 (0.80)	0.293	n.s
Fluency domain	0.35 (1.14)	− 0.04 (1.37)	− 0.20 (0.55)	0.496	n.s
Verbal memory domain	0.05 (1.03)	0.21 (1.04)	− 0.17 (1.01)	0.491	n.s
Processing speed, switch, and control domain	0.10 (0.86)	− 0.08 (1.13)	− 0.01 (1.07)	0.954	n.s
Switching domain	− 0.37 (1.29)	0.27 (0.72)	0.07 (0.95)	0.522	n.s

Means and standard deviations are displayed. Abbreviations: eFSIQ = estimated age-standardized full-scale intelligence quotient

^a^ *p*-values and Cohen’s d effect sizes for treatment group comparisons using MANCOVA are provided

Age, sex and parental educational level were added as covariates to all analyses

### Association between brain structure and neurocognitive functioning in severe CKD patients

**Fig. 1:**
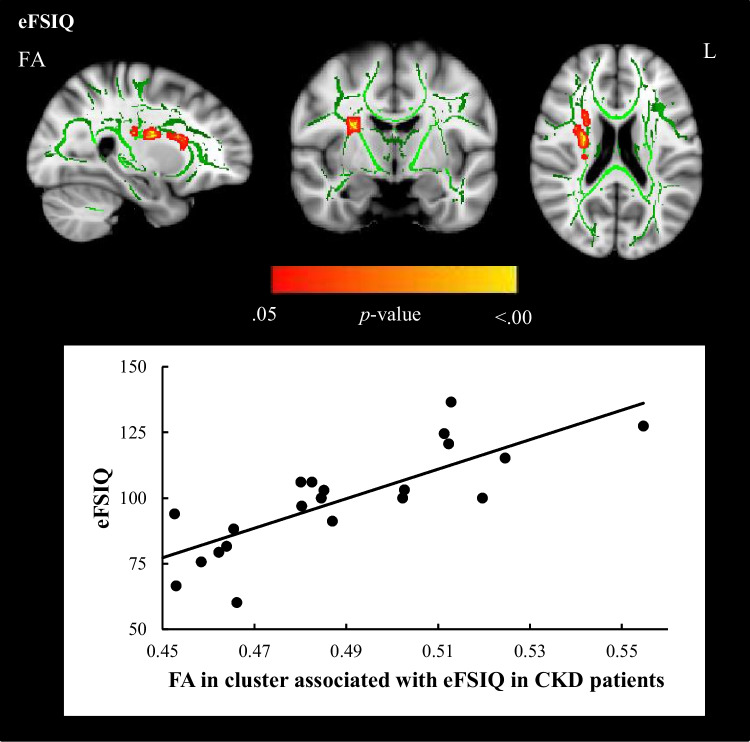
Regional associations between FA in cluster affected by CKD and eFSIQ using threshold-free cluster enhancement correction in TBSS, showing a significant positive correlation between eFSIQ and white matter integrity in CKD patients (displayed in red–yellow, *n* = 23, sociodemographic characteristics were included in the model as covariates). Image is illustrated the following coordinates: (*x* = 26, *y* =  − 7, *z* = 20) and shows the whole brain skeleton (at FA > 0.3, in green), overlaid on standard MNI 152 1-mm T1 brain. Significant group differences are “thickened” towards the full width of the white matter tract to increase visualization. The scatterplot shows the association between eFSIQ and FA in the cluster that is associated with eFSIQ in the CKD sample. Abbreviations: FA, fractional anisotropy; L, left

Regional associations between FA and neurocognitive functions with observed effects of CKD revealed a cluster of white matter tracts in the whole CKD group (complete MRI and neurocognitive data, *n* = 23), in which lower FA was associated with lower eFSIQ (Fig. 1). This cluster of tracts involves the SLF, CST, ATR, and IFOF. No regional associations between FA and processing speed and working memory were observed. The cluster of white matter tracts relating to eFSIQ in CKD patients was selected for further analyses investigating the relation between brain structure and neurocognitive functioning.

Univariate analyses on eFSIQ showed *p*-values < 0.20 for FA in the selected cluster of white matter tracts (Supplement 4). The final multivariate regression analyses revealed that lower FA in the selected cluster of white matter tracts and lower parental education level were significantly related to lower eFSIQ. Univariate analyses on processing speed and working memory showed *p*-values < 0.20 for FA in the selected cluster of white matter tracts. The final multivariate regressions analyses revealed that older age and lower parental educational level were significantly related to worse processing speed and working memory performance (Table 5).

**Table 5:** Multivariate regression analyses on associations between CKD-related brain abnormalities and neurocognitive functioning

Outcome variable	Predictors retained in the model	Statistics			Model performance		
		*B* (*SE*)	*β*	*P*	*F*	*R*^*2*^	*p*
eFSIQ	Parental educational level	4.92 (2.27)	0.258	0.042	28.34	0.739	< 0.001
	FA within the cluster of CKD-affected white matter tracts associated with eFSIQ	513.11 (93.48)	0.751	< 0.001			
Processing speed and working memory	Age (years)	− 0.07 (0.03)	− 0.450	0.013			
	Parental educational level	0.78 (0.28)	0.466	0.011	8.35	0.455	0.002

MRI data and neurocognitive data were available for a subsample of CKD patients (*n* = 23) [10]. Age, sex, and parental educational level were added as covariates to all analyses

*Abbreviations*: CKD = chronic kidney disease, eFSIQ = estimated age-standardized full-scale intelligence quotient, FA = fractional anisotropy, SE = standard error

**Fig. 2:**
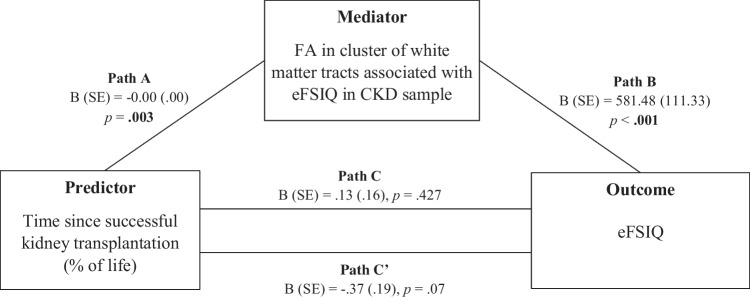
Mediation model testing the impact of FA in the cluster of white matter tracts associated with eFSIQ in CKD patients in the association between time since successful kidney transplantation (% of life) and eFSIQ. Age, sex, and parental educational level were added as covariates to mediation analyses. Illustration of the indirect (paths A and B), direct (path C) and total effects (path C′), showing that the effect of time since successful kidney transplantation on eFSIQ is mediated by FA in the cluster of white matter tracts associated with eFSIQ. *Abbreviations:* FA = fractional anisotropy; B = unstandardized regression coefficient; eFSIQ = estimated full-scale intelligence quotient; SE = standard error

Mediation analysis revealed that lower FA in the selected cluster of white matter tracts significantly mediated the relation between time since successful kidney transplantation and eFSIQ (95% confidence interval =  − 0.92 to − 0.15; Fig. 2).

## Discussion

In this study, we explored the impact of a selection of clinical risk factors on neurocognitive functioning and the relation with structural brain abnormalities in children and young adults with severe CKD. We found that greater exposure to dialysis, longer time since transplantation and later onset of severe CKD are risk factors of worse neurocognitive outcomes (i.e., intelligence and/or processing speed and working memory). In addition, the association between longer time since transplantation and lower intelligence was mediated by regional disruption of white matter integrity. The results of this study suggest that the occurrence of white matter abnormalities in young patients with advanced stages of CKD may play an instrumental role in the development of neurocognitive impairment.

We found no evidence for a direct impact of low eGFR or duration of severe CKD on neurocognitive functioning. Although planned analyses initially showed no group differences between treatment subgroups, exploratory analysis suggests that patients on KRT (transplanted and dialysis patients) may have worse neurocognitive outcome than pre-KRT patients with an eGFR below 30 ml/min/1.73 m^2^. This gives rise to the hypothesis that (a history of) prolonged duration of KRT may be more strongly related to neurocognitive functioning than exposure to uremic toxins. This hypothesis remains to be further investigated, but is in line with previous studies showing that neurocognitive functioning of transplanted children is impaired in comparison to healthy peers [2] and that neurocognitive functions are sensitive to the impact of dialysis in both pediatric and adult patients [2, 4, 29]. Our finding that greater exposure to the state of kidney transplantation is associated with lower intelligence contrasts with outcomes from adult CKD studies, which show that neurocognitive functioning of adults with a well-functioning kidney graft is not impaired relative to healthy controls [4]. Together, these potentially worrying findings would imply that the course of CKD and transplantation may have a different and more negative impact on neurocognitive functioning of young patients compared to adult patients. Our finding that patients with an older age at severe CKD diagnosis have worse neurocognitive functioning regarding processing speed and working memory is surprising and needs to be confirmed in future studies. However, it could be speculated that early reorganization (i.e., neuroplasticity) in the younger brain may (partly) compensate the initial impact of CKD on processing speed and working memory. Finally, no evidence was found for an impact of severe CKD on other neurocognitive domains (e.g., fluency, verbal memory, cognitive control and switching), which somewhat contrasts with previous studies in children and adult patients [2, 4]. Although our study may have lacked power to detect subtle effects of severe CKD on specific neurocognitive domains, this discrepancy may also be explained by differences in assessment methodology which may subvert direct comparisons between study outcomes. Furthermore, previous claims that children with CKD are at risk for executive function problems have often been based on patient and proxy report questionnaires [2], which are known to measure a different construct than performance-based assessments of executive functioning [30].

Our analyses also suggest that disrupted white matter integrity in specific tracts (i.e., SLF, CST, ATR, and IFOF) is related to lower intelligence in our severe CKD patients. This partly contrasts with a previous study in children with mild to moderate stages of CKD where no evidence for an association between CKD-related white matter integrity and neurocognitive functioning was found [18], suggesting that abnormal white matter integrity may have stronger relevance to neurocognitive functioning in more advanced stages of CKD than in milder stages of CKD. This implies that advanced CKD may be associated with abnormal development of white matter tracts, possibly causing less efficient information processing within brain neural networks that are essential for higher-order functions, such as intelligence [31, 32]. Indeed, lower white matter integrity has previously been linked to worse global neurocognitive functioning in adults with CKD [12, 14]. Importantly, we found a mediating role for white matter disruption in the relation between time since transplantation and lower intelligence. This finding may suggest that CKD-related disease mechanisms disrupt white matter integrity in young patients, subsequently contributing to neurocognitive impairments.

To our knowledge, this is the first study to report a mediating role of white matter integrity in neurocognitive outcome of CKD patients and this observation awaits replication in longitudinal studies. The association with time since transplantation may reflect that the initial impact of CKD on the developing brain and intelligence may continue after transplantation. Additionally, from this finding, it could be speculated that factors post-transplantation (e.g., impaired microcirculation, neurotoxic and vasoactive immunosuppressive therapy) may have further detrimental effects on brain structure and neurocognitive functioning [7–9]. The idea that an impaired microcirculation (e.g., due to hypertension or vaso-active medication) is implicated in the development of white matter abnormalities and associated neurocognitive impairments post-transplantation is consistent with previous work showing that the burden of cardiovascular disease increases with longer time since transplantation [33] and that cardiovascular risk factors are both related to lower white matter integrity and neurocognitive problems [34, 35].

Taken together, we argue that our findings reflect a complex pathogenesis of CKD-related brain abnormalities that may comprise both disease and treatment (i.e., transplantation and dialysis) -related factors. These factors may lead to white matter disruption and subsequent neurocognitive impairment, which may not fully recover after transplantation.

## Limitations and strengths

First, we acknowledge our small sample size. Therefore, cautious interpretation of our findings is necessary. We encourage further investigation of neurocognitive functioning and underlying neuropathology in dialysis and transplanted patients using prospective, long-term longitudinal designs with multivariate repeated measurements within individual patients to increase rigor of the observations and to follow the course of CKD throughout disease and treatment stages. We cannot exclude that other factors may have influenced neurocognitive development in conjunction with CKD, such as prematurity, perinatal events, and genomic disorders that are prevalent co-occurring diagnoses in this population and have previously been associated with neurocognitive impairments [36]. It is important that future research in larger cohorts take into account the potential roles of co-morbidities and clinical complications of CKD (e.g., hypertension, prematurity, prolonged severe acidosis, proteinuria, disease etiology). A second limitation is the lack of a matched control group. Although we compared neurocognitive functioning of our CKD patients to normative data, it is necessary to replicate these findings and compare patients to a matched healthy control group. A third limitation is the heterogeneity of the sample in terms of socio-demographic and illness characteristics. Sociodemographic variables were included as covariates in statistical analyses to take possible confounding factors into account, to partly account for heterogeneity. This study also has several strengths. It is the first study to focus on the association between CKD-related white matter abnormalities and neurocognitive functioning in a representative sample of young Dutch-speaking patients with advanced CKD on different treatment modes, using an extensive neuropsychological test battery, relate this to brain structure (using advanced quantitative analyses), and perform mediation analyses. Thereby, this study contributes to our understanding of the disease mechanisms and neural mechanisms that underlie neurocognitive impairment in young patients with severe CKD.

## Clinical implications and future directions

Our findings highlight the importance for clinicians to be aware of neurocognitive problems in children and young adults with advanced CKD, in particular patients who have a complex medical history of prolonged time on KRT. As compared to pre-KRT patients, patients on dialysis as well as transplanted patients seem to be at risk for neurocognitive impairment. When clinicians have concerns on neurocognitive functioning of a child or young adult with CKD, it is recommended to conduct neuropsychological assessment to identify possible neurocognitive impairments to determine which patients may benefit from (neuro)psychological support.

This is the first study to indicate a mediating role of CKD-associated brain white matter abnormalities in the development of neurocognitive impairments in young severe CKD patients, which may provide a potential target for the development of neurocognitive rehabilitation strategies. Physical exercise is suggested to indirectly improve neurocognitive functioning in healthy adolescents and young adults through a possible beneficial impact on white matter integrity (i.e., through the increase of neurotrophic factors and cerebral blood flow) [37–40]. This suggests an opportunity for physical exercise as a neurocognitive rehabilitation strategy, although effectiveness in clinical populations remains to be determined [41].

Finally, it is important that prospective longitudinal studies further determine the effects of dialysis and kidney transplantation on neurocognitive functioning and underlying neural mechanisms (e.g., using functional brain connectivity and/or measuring biomarkers of neuroaxonal injury) as well as investigating the impact of worse neurocognitive functioning on health-related quality of life and adaptive functioning.

## Supplementary information

Below is the link to the electronic supplementary material.

Graphical Abstract (PPTX 796 KB)

Supplementary file2 (PDF 273 KB)

Supplementary file3 (PDF 274 KB)

Supplementary file4 (PDF 256 KB)

Supplementary file5 (PDF 256 KB)

## Abbreviations

ATR: Anterior thalamic radiation
ANOVA: Analysis of variance
CKD: Chronic kidney disease
CST: Corticospinal tract
eFSIQ: Estimated age-standardized full-scale intelligence quotient
eGFR: Estimated glomerular filtration rate
FA: Fractional anisotropy
IFOF: Inferior fronto-occipital fasciculus
MD: Mean diffusivity
MRI: Magnetic resonance imaging
Non-KRT: Non-kidney replacement therapy
KRT: Kidney replacement therapy
ROI: Region of interest
SLF: Superior longitudinal fasciculus
