# Supplementary material for: Risk factors for neurocognitive impairment and the relation with structural brain abnormality in children and young adults with severe chronic kidney disease
Source: Pediatr Nephrol. 2022 Nov 2;38(6):1957–69. doi: 10.1007/s00467-022-05781-1 (PMC10154258; doi:10.1007/s00467-022-05781-1)
Supplement: Supplementary file 1 — Graphical Abstract (PPTX 796 KB) [file 467_2022_5781_MOESM1_ESM.pptx]

## Slide 1
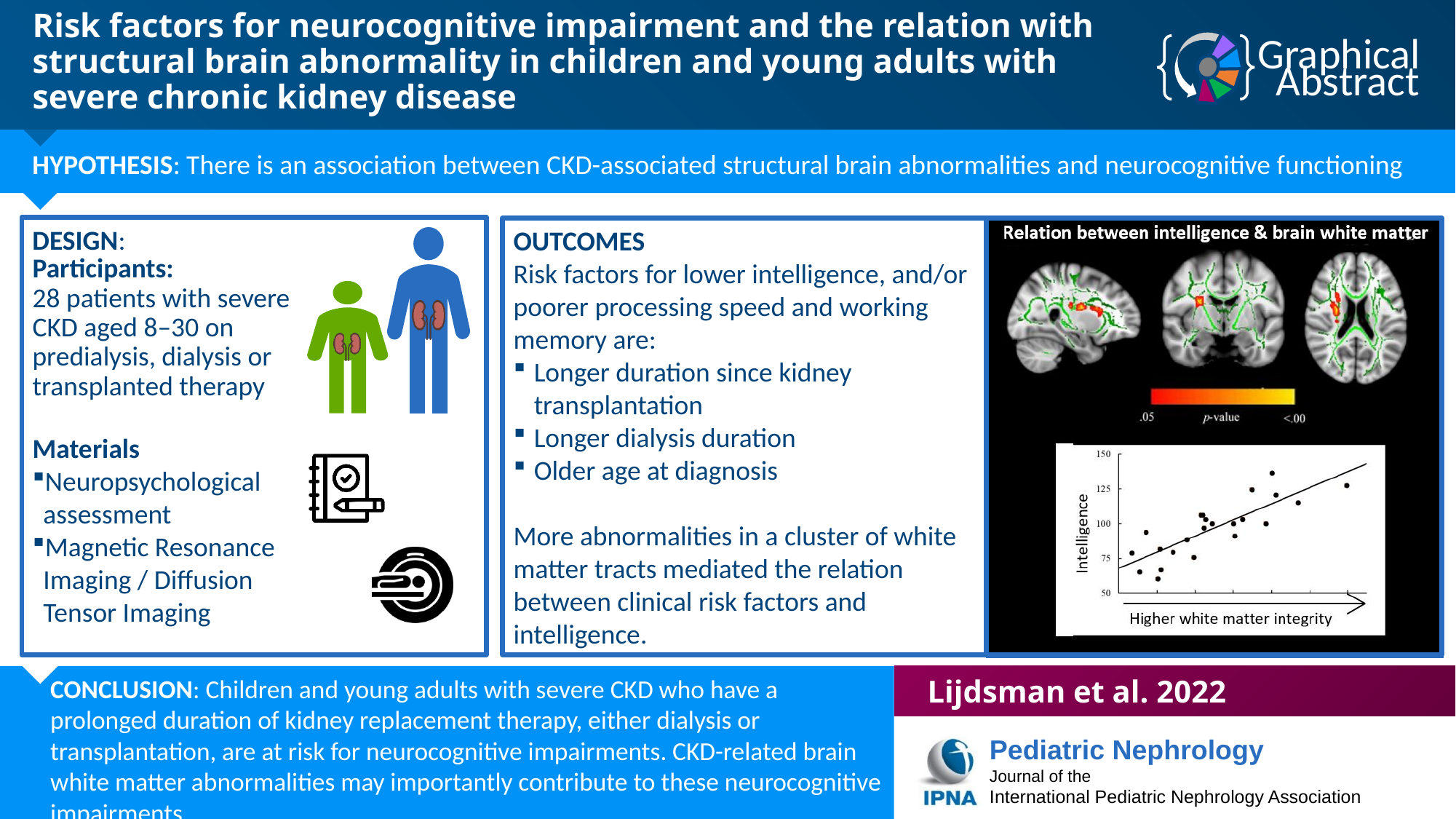

Risk factors for neurocognitive impairment and the relation with structural brain abnormality in children and young adults with severe chronic kidney disease
HYPOTHESIS: There is an association between CKD-associated structural brain abnormalities and neurocognitive functioning
DESIGN:
OUTCOMES
Risk factors for lower intelligence, and/or poorer processing speed and working memory are:
Longer duration since kidney transplantation
Longer dialysis duration
Older age at diagnosis
More abnormalities in a cluster of white matter tracts mediated the relation between clinical risk factors and intelligence.
Participants:
28 patients with severe CKD aged 8–30 on predialysis, dialysis or transplanted therapy
Materials
Neuropsychological assessment
Magnetic Resonance Imaging / Diffusion Tensor Imaging
CONCLUSION: Children and young adults with severe CKD who have a prolonged duration of kidney replacement therapy, either dialysis or transplantation, are at risk for neurocognitive impairments. CKD-related brain white matter abnormalities may importantly contribute to these neurocognitive impairments.
Lijdsman et al. 2022
