# Supplementary material for: Risk factors for neurocognitive impairment and the relation with structural brain abnormality in children and young adults with severe chronic kidney disease
Source: Pediatr Nephrol. 2022 Nov 2;38(6):1957–69. doi: 10.1007/s00467-022-05781-1 (PMC10154258; doi:10.1007/s00467-022-05781-1)
Supplement: Supplementary file 2 — Supplementary file2 (PDF 273 KB) [file 467_2022_5781_MOESM2_ESM.pdf]

Supplement 2.

*Univariate regression analyses on associations between CKD parameters and neurocognitive functioning*

| Outcome variable | Predictors | Statistics | | |
|---|---|---|---|---|
| | | B (SE) | *β* | *P* |
| eFSIQ | Age at CKD diagnosis (years) | -.01 (.51) | -.002 | -.991 |
| | eGFR (ml/min/1.73 $m^2$) | -.18 (.17) | -.198 | .313 |
| | Duration severe CKD (% of life) | -.12 | -.122 | .536 |
| | Dialysis duration (% of life) | -1.88 (.70) | -.464 | **.013** |
| | Time since kidney transplantation (% of life) | -.27 (.18) | -.288 | **.138** |
| Processing Speed & Working Memory | Age at CKD diagnosis (years) | -.05 (.02) | -.364 | **.057** |
| | eGFR (ml/min/1.73 $m^2$) | .00 (.01) | .048 | .810 |
| | Duration severe CKD (% of life) | .01 (.01) | .135 | .494 |
| | Dialysis duration (% of life) | -.13 (.03) | -.653 | **<.001** |
| | Time since kidney transplantation (% of life) | .00 (.01) | .022 | .913 |
| Verbal Fluency | Age at CKD diagnosis (years) | .01 (.03) | .039 | .846 |
| | eGFR (ml/min/1.73 $m^2$) | -.01 (.01) | -.209 | .287 |
| | Duration severe CKD (% of life) | .00 (.01) | .105 | .595 |
| | Dialysis duration (% of life) | .01 (.04) | .050 | .800 |
| | Time since kidney transplantation (% of life) | -.00 (.01) | -.058 | .771 |
| Verbal Memory | Age at CKD diagnosis (years) | .01 (.03) | .062 | .755 |

| | | | | |
|---|---|---|---|---|
| | eGFR (ml/min/1.73 $m^2$) | -.00 (.01) | -.079 | .691 |
| | Duration severe CKD (% of life) | -.02 (.01) | -.348 | **.069** |
| | Dialysis duration (% of life) | -.07 (.04) | -.358 | **.061** |
| | Time since kidney transplantation (% of life) | -.00 (.01) | -.037 | .851 |
| Processing Speed, Switch & Control | Age at CKD diagnosis (years) | .02 (.02) | .171 | .385 |
| | eGFR (ml/min/1.73 $m^2$) | .01 (.01) | .161 | .412 |
| | Duration severe CKD (% of life) | -.01 (.01) | -.220 | .260 |
| | Dialysis duration (% of life) | -.04 (.04) | -.217 | .268 |
| | Time since kidney transplantation (% of life) | -.00 (.01) | -.057 | .773 |
| Switching | Age at CKD diagnosis (years) | .02 (.02) | .191 | .329 |
| | eGFR (ml/min/1.73 $m^2$) | .01 (.01) | .198 | .312 |
| | Duration severe CKD (% of life) | -.01 (.01) | -.120 | .544 |
| | Dialysis duration (% of life) | -.03 (.04) | -.146 | .459 |
| | Time since kidney transplantation (% of life) | -.01 (.01) | -.163 | .406 |

*Note.* Statistical values p <.200 are shown in bold and these predictors were selected for further multivariate regression analyses. Abbreviations: CKD = Chronic kidney disease; eFSIQ = Estimation of age-standardized full-scale Intelligence Quotient; eGFR = estimated glomular filtration rate; SE = Standard Error.
