# Supplementary material for: Risk factors for neurocognitive impairment and the relation with structural brain abnormality in children and young adults with severe chronic kidney disease
Source: Pediatr Nephrol. 2022 Nov 2;38(6):1957–69. doi: 10.1007/s00467-022-05781-1 (PMC10154258; doi:10.1007/s00467-022-05781-1)
Supplement: Supplementary file 3 — Supplementary file3 (PDF 274 KB) [file 467_2022_5781_MOESM3_ESM.pdf]

Supplement 2.

Univariate regression analyses on associations between CKD parameters and neurocognitive functioning

Outcome variable	Predictors retained in the model	Statistics		
		B (SE)	*β*	*P*
eFSIQ	Age at CKD diagnosis (years)	-.01 (.51)	-.002	-.991
	eGFR (ml/min/1.73 m^2)	-.18 (.17)	-.198	.313
	Duration severe CKD (% of life)	-.12	-.122	.536
	Dialysis duration (% of life)	-1.88 (.70)	-.464	**.013**
	Time since kidney transplantation (% of life)	-.27 (.18)	-.288	**.138**
Processing Speed & Working Memory	Age at CKD diagnosis (years)	-.05 (.02)	-.364	**.057**
	eGFR (ml/min/1.73 m^2)	.00 (.01)	.048	.810
	Duration severe CKD (% of life)	.01 (.01)	.135	.494
	Dialysis duration (% of life)	-.13 (.03)	-.653	**<.001**
	Time since kidney transplantation (% of life)	.00 (.01)	.022	.913
Verbal Fluency	Age at CKD diagnosis (years)	.01 (.03)	.039	.846
	eGFR (ml/min/1.73 m^2)	-.01 (.01)	-.209	.287
	Duration severe CKD (% of life)	.00 (.01)	.105	.595
	Dialysis duration (% of life)	.01 (.04)	.050	.800
	Time since kidney transplantation (% of life)	-.00 (.01)	-.058	.771

Verbal Memory	Age at CKD diagnosis (years)	.01 (.03)	.062	.755
	eGFR (ml/min/1.73 m^2)	-.00 (.01)	-.079	.691
	Duration severe CKD (% of life)	-.02 (.01)	-.348	**.069**
	Dialysis duration (% of life)	-.07 (.04)	-.358	**.061**
	Time since kidney transplantation (% of life)	-.00 (.01)	-.037	.851
Processing Speed, Switch & Control	Age at CKD diagnosis (years)	.02 (.02)	.171	.385
	eGFR (ml/min/1.73 m^2)	.01 (.01)	.161	.412
	Duration severe CKD (% of life)	-.01 (.01)	-.220	.260
	Dialysis duration (% of life)	-.04 (.04)	-.217	.268
	Time since kidney transplantation (% of life)	-.00 (.01)	-.057	.773
Switching	Age at CKD diagnosis (years)	.02 (.02)	.191	.329
	eGFR (ml/min/1.73 m^2)	.01 (.01)	.198	.312
	Duration severe CKD (% of life)	-.01 (.01)	-.120	.544
	Dialysis duration (% of life)	-.03 (.04)	-.146	.459
	Time since kidney transplantation (% of life)	-.01 (.01)	-.163	.406

Note. Abbreviations: CKD = Chronic kidney disease; eFSIQ = Estimation of age-standardized full-scale Intelligence Quotient; eGFR = estimated glomular filtration rate; SE = Standard Error.
