# Supplementary material for: Risk factors for neurocognitive impairment and the relation with structural brain abnormality in children and young adults with severe chronic kidney disease
Source: Pediatr Nephrol. 2022 Nov 2;38(6):1957–69. doi: 10.1007/s00467-022-05781-1 (PMC10154258; doi:10.1007/s00467-022-05781-1)
Supplement: Supplementary file 5 — Supplementary file5 (PDF 256 KB) [file 467_2022_5781_MOESM5_ESM.pdf]

Supplement 4.

Univariate regression analyses on associations between aspects of brain structure and neurocognitive functioning

Outcome variable	Predictors retained in the model	Statistics		
		B (SE)	*β*	*P*
eFSIQ	Nucleus Accumbens volume (cm^3)	.00 (.00)	.085	.691
	FA within the cluster of CKD-affected white matter tracts associated with eFSIQ	562.16 (84.61)	.823	**<.001**
	MD within the cluster of CKD-affected white matter tracts associated with eFSIQ (10^{-}5 mm2/s)	-.41175.51 (169798.84)	-.053	.811
Processing Speed & Working Memory	Nucleus Accumbens volume (cm^3)	.00 (.00)	.090	.675
	FA within the cluster of CKD-affected white matter tracts associated with eFSIQ	12.43 (6.77)	.372	**.080**
	MD within the cluster of CKD-affected white matter tracts associated with eFSIQ (10^{-}5 mm2/s)	-5664.24 (8230.35)	-.149	.499

Note. Abbreviations: CKD = Chronic kidney disease; eFSIQ = Estimation of age-standardized full-scale Intelligence Quotient; FA = fractional anisotropy; MD = mean diffusivity; SE = Standard Error.
